# Deepening the understanding of social bonding and dynamics of social interactions

**DOI:** 10.1111/gbb.12804

**Published:** 2022-03-09

**Authors:** Andrey E. Ryabinin

**Affiliations:** ^1^ Department of Behavioral Neuroscience Oregon Health and Science University Portland Oregon USA

**Keywords:** dominance, fire ant, oxytocin, prairie vole, social affiliation, social bonding, social hierarchies, zebra finch

The current volume continues the series of Genes, Brain and Behavior special issues. In the first special issue, we focused on autism and neurodevelopmental disorders.^1^ We concluded the issue with the idea that detailed understanding of these disorders is impossible without profound appreciation of mechanisms underlying natural species‐specific social behaviors. Therefore, the current special issue directs readers' attention to fundamentals of social bonding, interactions, group/colony formation and even potential biology of human sociocultural experiences.

We start with social bonding, or social attachment. This topic has been not only an extensive subject of poetry and fictional literature, but has also been examined across multiple scientific disciplines, including psychology, anthropology, ethology, animal behavior, and cognitive neuroscience. Each of the disciplines provided explanations of this phenomenon in their own terms. A synthesis of these terms would be a substantial intellectual achievement. This need for synthesis is the cornerstone of the review by Gustison and Phelps,^2^ the first article in this issue. The authors explore views on social attachment across multiple disciplines and then focus on pair‐bond formation in prairie voles as a case study. The prairie vole (*Microtus ochrogaster)* is a socially monogamous rodent species. Studies of these species provided much insight into mechanisms of social bonding.^3^, ^4^ The authors describe the intricate molecular and circuitry mechanisms involved in individual differences in social bonding in prairie voles and provide an example of integration of this knowledge into multi‐disciplinary language.

The next article, a review by Prior et al.^5^ provides another type of integration of our understanding of mechanisms involved in social bonding. Specifically, the authors examine how much the social aspects of bond formation are dependent on the involved sensory and perceptual systems. They use the prairie vole and the zebra finch as examples of species, in which neurocircuitry of either chemical or vocal communication, and of social bonding, have been examined. The authors discuss the inter‐dependence and bidirectional influences between specific sensory processes and social bonding. This exploration is followed by a framework for future studies in this field.

The reviews mentioned above are followed by two companion research papers, which challenge the simplified views on processes involved in pair bonding of prairie voles. They provide critical evidence against the idea of potential monomorphism in pair‐bonding behaviors in these socially monogamous species. The article by Brusman et al.^6^ compares indices of pair‐bonding in the standard “choice” partner preference test^7^, ^8^ and a novel modified non‐choice test across two time points between male and female prairie voles. Using the non‐choice test allows authors to document that the increased interaction with the partner versus a novel (“stranger”) vole is driven by female animals. A subsequent social operant version of the procedure expands on this observation by showing that motivation to access a partner is significantly stronger in females than in males.

In the companion research paper by Vahaba et al.,^9^ authors use their own version of operant social choice task to provide further insight into sex differences in pair bonding in prairie voles. They demonstrate that female voles are willing to work hardest to gain access to their mate versus an unfamiliar male. In contrast, males on average lever‐press equally for either their partner or “stranger” female vole. Importantly, this average lack of preference for their mate is not due to potential lack of preference across all males, but is driven by characteristic partner‐directed, stranger‐directed or indifferent behavior of individual male voles. This observation calls for genetic analysis of differences driving bonding‐associated behaviors, which the authors initiate by genotyping the oxytocin receptor gene. Thus, the research papers for Brusman et al. and Vahaba et al. provide striking examples of how a presumably sexually monomorphic social bonding behavior in prairie voles is driven by dimorphic motivational aspects of this behavior.

Our next article departs from analysis of social bonding in mammals and birds to examine mechanisms of plasticity involved in formation of groups of individuals in a completely different taxon. Namely, Manfredini et al.^10^ examine molecular signatures of plasticity associated with differences in social environment in the fire ants (*Solenopsis invicta*). Queens of this species can found colonies in either solitary or group conditions.^11^ The researchers examine global co‐expression networks in the brain of these queens at two stages of colony founding when they are placed in either solitary or in group conditions. Group conditions of two sizes are examined. Increasing the group sizes results in larger changes in gene expression. The findings indicate that important plasticity changes are associated not only with simple solitary versus groups housing differences but can be also quite striking when associated with apparent subtle differences in population size.

It is well appreciated that social relationships and interactions are not uniform across individuals within one species. Differences in these interactions can be reflected in social hierarchies. There has been notable interest in examining neural mechanisms contributing to development of dominant versus submissive social status of individuals within species.^12^ However, methods for examining this status vary and can produce varied results across different species and sexes. In their review, Fulenwider et al. examine different methodologies for evaluating social rank across rodent species.^13^ The methodologies are either based on the extent of agonistic behaviors that individual animals exhibit or experience, or on the differences in access to valuable resources by these individuals. Species and sex differences, as well as pitfalls and understudied research areas, are discussed.

The volume concludes with an article highlighting relationships between molecular mechanisms regulating social behaviors and sociocultural experiences in humans. Lee et al. present a gene‐culture coevolution case study of associations between polymorphisms in the oxytocin receptor gene and cultural tightness and socio‐ecological threats across human populations.^14^ The authors find that cultural tightness and socio‐ecological threats are positively correlated with polymorphisms regulating expression of this receptor in the anterior cingulate cortex. These findings provocatively suggest that polymorphisms driving enhanced oxytocin receptor expression underwent positive selection in human populations with tight cultural norms.

Taken the discussed articles together, as expected, social interactions are governed by exhilaratingly complex multilevel mechanisms. Species differences, sex differences, and individual differences contribute to this complexity. These differences need to be appreciated when examining mechanisms of mental disorders. In the next special issue in this series of Genes, Brain and Behavior, we will continue to present articles on natural social behaviors and transition into models of mental disorders associated with changes in social behaviors.

## Data Availability

Data sharing is not applicable to this article as no new data were created or analyzed in this study.
